# Heart Failure Syndrome With Preserved Ejection Fraction Is a Metabolic Cluster of Non-resolving Inflammation in Obesity

**DOI:** 10.3389/fcvm.2021.695952

**Published:** 2021-08-02

**Authors:** Bochra Tourki, Ganesh V. Halade

**Affiliations:** Division of Cardiovascular Sciences, Department of Medicine, The University of South Florida, Tampa, FL, United States

**Keywords:** HFPEF, immune metabolism, endothelial dysfunction, macrophages, obesity

## Abstract

Heart failure with preserved ejection fraction (HFpEF) is an emerging disease with signs of nonresolving inflammation, endothelial dysfunction, and multiorgan defects. Moreover, based on the clinical signs and symptoms and the rise of the obesity epidemic, the number of patients developing HFpEF is increasing. From recent molecular and cellular studies, it becomes evident that HFpEF is not a single and homogenous disease but a cluster of heterogeneous pathophysiology with aging at the base of the pyramid. Obesity superimposed on aging drives the number of inflammatory pathways that intersect with metabolic dysfunction and suboptimal inflammation. Here, we compiled information on obesity-directed macrophage dysfunction that coincide with metabolic defects. Obesity-associated proinflammatory stimuli facilitates heart and interorgan inflammation in HFpEF. Furthermore, diversified mechanisms that drive heart failure urge the need of studying pervasive and unresolved inflammation in animal models to understand HFpEF. A broad and system-based approach will help to study major translational aspects of HFpEF, since no single animal model recapitulates all signs of differential HFpEF stages in the clinical setting. Here, we covered experimental models that target HFpEF and emphasized the advances observed with formyl peptide 2 (FPR2) receptor, a prime sensor that is important in inflammation-resolution signaling. Dysfunction of FPR2 led to the development of spontaneous obesity, impaired macrophage function, and triggered kidney fibrosis, providing evidence of multiorgan defects in HFpEF in an obesogenic aging experimental model.

## Evolution of Multidimensional Metabolic Heart Failure Within the Obesity Epidemic

Obesity serves as an incubator for many cardiometabolic and cardiorenal defects. Hypertension, insulin resistance, diabetes, and dysplasia are major metabolic diseases that are strongly linked to obesity. The prevalence of metabolic defects is exponentially rising parallel to the trend of obesity epidemic (1). Epidemiological reports indicate the development of metabolic syndrome that is documented based on body mass with fat localization in intra-abdominal sites, including ectopic fat in the liver, pancreas, and heart (2).

In this minireview, first, we highlight inflammation-mediated pathogenesis in the emerging heart failure with preserved ejection fraction (HFpEF) syndrome. Second, we cover the concept of non-resolving and multiorgan chronic inflammation with an updated list of experimental animal models of HFpEF syndrome.

**Figure 1:**
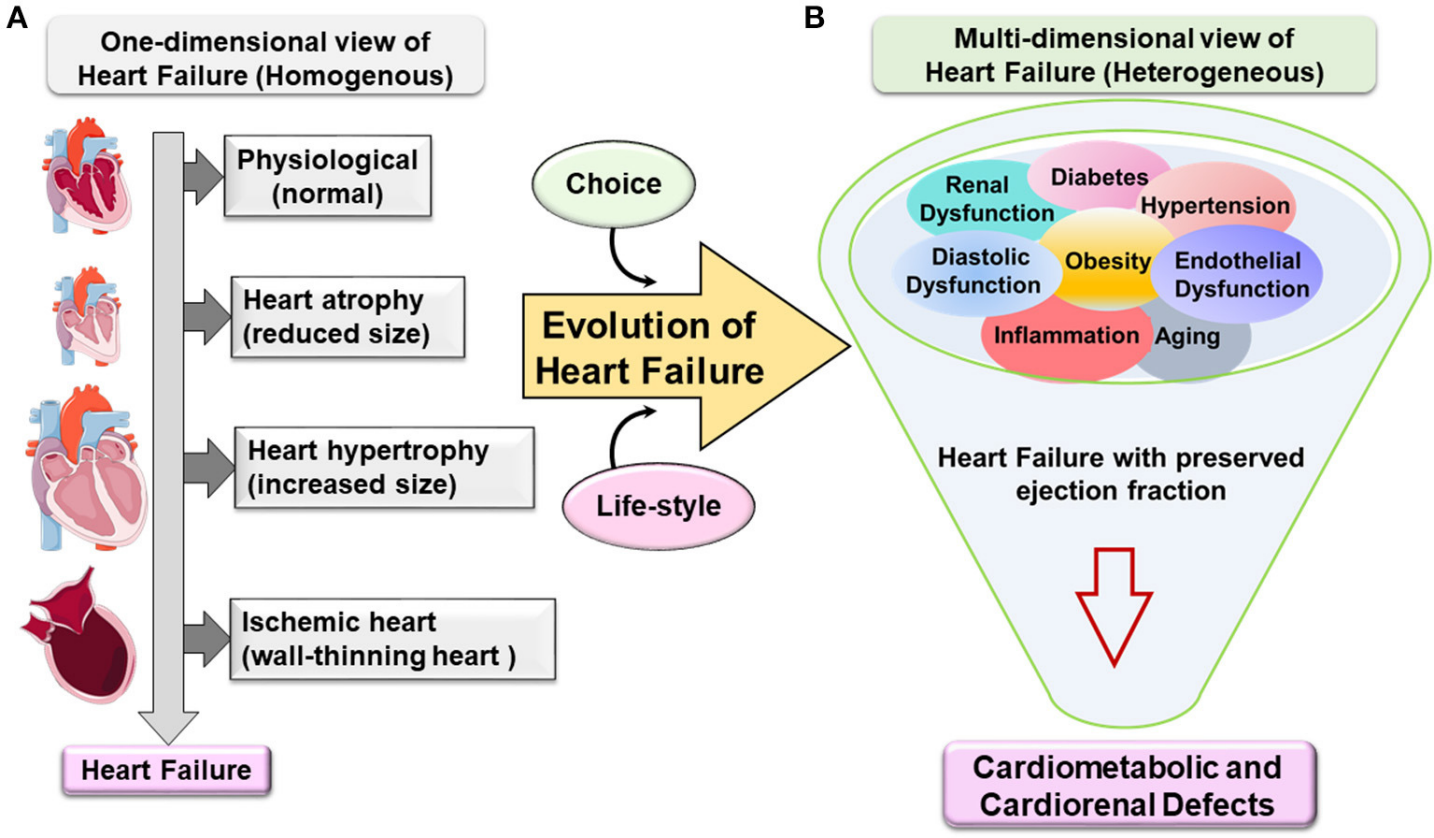
Evolution of one-dimensional to multidimensional view of cardiovascular disease and heart failure. **(A)** One-dimensional view of heart failure (HF). Classically, HF emerges in a homogenous population based on geometry, structure, and function. The progress from being a phenotypically normal to abnormal structure accompanied with a decrease or increase in the (cardiomyocyte) heart size due to eccentric or concentric remodeling or following ischemic injury with dilation of the chambers and wall-thinning effect. HF specifically with preserved ejection fraction evolves from one-dimensional view to multidimensional view. **(B)** Metabolic defects directed the multidimensional view of heart failure with preserved ejection fraction (HFpEF). Heterogeneous metabolic comorbidities are a primary confounder in HFpEF patients like obesity, hypertension, kidney dysfunction, diabetes, aging, etc. The complexity of the HFpEF syndrome resulted in cardiometabolic and cardiorenal defects with signs of suboptimal inflammation without any current treatment.

In the last five decades, epidemiological and systemic research helped in clarifying that obesity is closely associated with multiple changes in the structure and function of the heart of obese individuals. Clinicians and scientists described HF as a pathological remodeling of left ventricle (LV) cardiomyocytes. This remodeling is a result of heart adaptation to blood hemodynamics along with the increase in fat mass accumulation. With the advances in ultrasound and magnetic resonance imaging techniques, a clear evolution of LV shape remodeling was observed ranging from one-dimensional view to multidimensional subtypes of HF with specific nomenclature that reflects the corresponding remodeling (Figure 1). Furthermore, in extreme obesity, the main contributors of end-stage HF are dysfunction of the right ventricle as well as prominent diastolic dysfunction with LV stiffness, lung edema, and low-grade and suboptimal systemic inflammation.

From the one-dimensional perspective, HF is classified into three types based on geometry metamorphosis of the LV: (1) atrophy of the heart (contractile dysfunction), (2) concentric hypertrophy of the LV (thick wall), and (3) eccentric hypertrophy of the LV (thin wall) or ischemia-induced wall thinning. However, some rare cases of cardiac angiosarcoma can also lead to HF (3, 4). This report emphasizes the evolution of HFpEF syndrome due to possible obesity-directed metabolic defects and multiorgan inflammation.

### Atrophy of the Heart

Atrophy of the aging heart is a consequence of sarcopenic heart remodeling following muscle wasting. In the elderly, the size of a tissue or organ decreases due to cellular shrinkage (5). The prevalence of sarcopenia in chronic HF patients amounts to up to 20% and may progress into cardiac cachexia. Muscle wasting is a strong predictor of frailty and reduced survival in HF patients (6). Basically, heart atrophy can lead to reduced cardiac workload; however, complex inflammatory disease can result also in heart atrophy (7). Cardiac dysfunction is the outcome of the molecular changes resulting from cardiac atrophy and fibrosis (8). Molecular and cellular signaling pathways that underlie heart atrophy are not fully understood. However, some putative mechanisms for cardiac atrophy were observed in cancer patients (9). In some cases, atrophy of the heart can also be observed as a consequence of oncological drug treatments. Experimental and clinical studies showed that anthracycline chemotherapeutics, such as doxorubicin, induced heart atrophy in patients and mice (10). Mice treated with doxorubicin developed myocardial fibrosis, displayed splenic contraction, increased myocyte apoptosis, and impaired pumping of the heart (11). This latter has been connected to the release of myostatin by the atrophic heart muscle, which induces muscle wasting in HF (12).

Cardiac atrophy can occur not only in pathological conditions (13) but also after prolonged horizontal bed rest that may occur after short-term space flights, due to a decrease in unload volume to less activity or to the impact of microgravity in astronauts (14). Recent clinical studies are trying to better understand the paradigm of how the heart shrinks, to become atrophic, but increases its strength as observed with exercise (15).

### Hypertrophy of the Heart

Enlargement of the left ventricle (LV) is an adaptive process that evolves into maladaptive remodeling, which has long been recognized as one of the prime dysfunction mechanisms leading to HF. The heart is an extremely plastic organ and changes its geometry to compensate for either pressure or volume overload, normally resulting from exercise or obesity-related mechanical impact. This compensation has often been viewed as a feedback loop (16). Concentric hypertrophy, which develops in pressure overload, normalizes wall stress. However, eccentric hypertrophy that develops in volume overload as a result of ischemic insult allows for an increase in total stroke volume leading to defective physiological regulation (17). Therefore, ejection fraction (EF) as a measurement of the heart capability of pushing out the blood to the organs has emerged as a clinically useful phenotypic marker, indicative of unique pathophysiological mechanisms (18) and, most importantly, as tangible response to therapies (19). As selection and classification criteria, patients with EF ≤40% are classified as HF with reduced EF; however, when EF is ≥50%, patients are diagnosed with preserved EF (20, 21). In simplified terms, there is no “single” straightforward clear cause or trigger of HF in patients. Several cellular and molecular pathways have been observed as direct or indirect consequence of the development or/and aggravation of the disease depending on the diverse root cause for HF (22). Due to sedentary lifestyle and lack of physical activity, different signs of HF are caused by defective fatty acid oxidation signaling and metabolic syndrome in the setting of obesity. At the molecular level, most observed changes in signaling pathways in cardiac hypertrophy relate to the decrease in PI3K–Akt signaling thereby inhibiting eNOS activation and its downstream signaling, which is reflected by endothelial dysfunction (23). Another common observed metabolism disturbance is the increase in PPARα activity, which simulates a cascade of transcriptional coactivators, such as peroxisome proliferator-activated receptor-α coactivator (PGC-1α) and peroxisome proliferator-activated receptor-β coactivator (PGC-1β), that form active transcriptional complexes with CREB-binding protein (CBP/p300) and steroid receptor coactivator 1 (SRC-1) (24, 25). Another cellular and molecular consequence of excess of fatty acid flux in HF patients is the increase in free radical production (26).

#### Heart Failure With Reduced Ejection Fraction

Even though LV geometry is at the center of heart remodeling in HF, the LV EF is taking over on clinical classification of HFrEF or HFpEF (27). Both forms of heart pathology are well-established and explained in previous reviews. Multiple original reports described the morphologic and the hemodynamic differences in animal models (28, 29) and clinical settings (17, 30, 31). The etiology of HFrEF is associated mostly with idiopathic dilated cardiomyopathy (DCM) and ischemic insult, resulting in systolic dysfunction of the heart (32). However, diastolic dysfunction is linked to HFpEF pathology (33). Commonly, HFrEF is a result of ischemic insult due to myocardial infarction (MI) caused by artery occlusion with signs of plaque formation. The plaque formation is an accumulation of lipid, especially cholesterol, infiltration of monocyte/macrophages, proliferation of smooth muscle cells, and accumulation of connective tissue components, thereby, forming a thrombus. This phenomena of atherogenesis depict a complicated stage of atheroprogression in the presence of inflammatory milieu as reported in obesity (34). After thrombosis, NO release is impaired, or there is a decrease in NO-(cGMP) signaling bioavailability to the cardiomyocytes. The decrease in NO is believed to be ultimately related to low-grade systemic inflammation and reduced cardiac perfusion (35–37). Despite the progress in the treatment of HFrEF (38), the initial point that leads to the development and progression of HFrEF is still convoluted, and more information is needed to understand the genesis of the disease (39, 40). From a metabolic point of view, abnormalities in myocardial energy metabolism in HFrEF have been reported but remain unclear due to the large number of metabolites/lipid mediators that control cardiac bioenergetics (41). At the cellular level, the disruption of electrical activity is present in both patients and animal models due to abnormalities in fat quality/quantity intake, sodium, and potassium channels on the myocardial membrane, which can lead to arrhythmias as well as cellular calcium dysregulation and altered calcium kinetics, thereby, leading to pathological heart contraction (42–45).

#### Heart Failure With Preserved Ejection Fraction

Technically, if the EF is preserved, evidence of altered cardiac structure and function should be sought to provide further objective evidence toward HF syndrome. At the functional level, Doppler echocardiographic evidence of diastolic dysfunction is appearing as a digital biopsy. This is reflected in echocardiography by slow ventricular relaxation and increased diastolic stiffness or elevated left atrial pressure, which is common in HFpEF (27) with multiple cardiometabolic defects (46); however, clear targets and their molecular mechanisms remain to be identified.

In the USA, more than 50% of HF patients have HFpEF with primary signs of obesity and heterogeneous metabolic syndrome (hypertension, insulin resistance, diabetes, and hyperlipidemia) (35, 47). In other words, the progress in the understanding of HF syndrome is not only based on structure/function of the heart but also includes heterogeneous arms of lifestyle-related triggers that promote HFpEF syndrome. As the etiology of HF syndrome is based on comorbidities and risk factors like aging, obesity, hypertension, and diabetes, the term multidimensional approaches needs to be considered in the development of preclinical HFpEF model and clinical treatment strategy (Figure 1) (48).

## Heart Failure Syndrome

HFrEF and HFpEF are end-stage disease pathologies with many confounding and overarching metabolic defects, thus, termed here as heart failure syndrome rather than disease. The pathophysiology of HFpEF is multifaceted signatures and heterogeneous, complex, and progressive. The increasing prevalence of HFpEF patients with aging and obesity defines a burden on healthcare systems (49). In the current clinical practice, HFpEF is reported as single disease abnormalities with LV diastolic dysfunction, chronotropic incompetence, and arterial stiffening (35). However, obesity or metabolic syndrome-driven endothelial dysfunction appeared as strongly related to HFpEF syndrome with multiorgan inflammation. Currently, obesity and inflammation are not considered as an epiphenomenon or prominent comorbidities for HFpEF but are intimately linked to its pathogenesis with progressive advancement (Figure 1) (50, 51). To recapitulate multiorgan etiologies, the development of accurate animal models is critical for the development of HFpEF targets (Table 1).

**Table 1:** List of current heart failure with preserved ejection fraction (HFpEF) animal models with coexistence of metabolic phenotype.

**Animal model**	**Phenotype**	**References**
FPR2KO	✓ Obesity ✓ Cardiac dysfunction ✓ Aging ✓ Renal dysfunction ✓ Systemic inflammation ✓ Immunometabolism dysfunction Hypertension? Diabetes?	Mouse (28, 52)
HFD+L-NAME	✓ Obesity ✓ Hypertensive stress ✓ Systemic inflammation ✓ Cardiac dysfunction ✓ Hypertension Aging? Renal dysfunction? Immunometabolism dysfunction?	Mouse (53)
Ob/ob or db/db	✓ Obesity ✓ Diabetes phenotype Aging? Renal dysfunction? Systemic inflammation? Immunometabolism dysfunction?	Mouse (54, 55)
ZF/ZDF	✓ Obesity ✓ Diabetes phenotype Cardiac dysfunction? Renal dysfunction? Aging? Systemic inflammation? Immunometabolism dysfunction?	Rat (56, 57)
SKO	✓ Diabetes phenotype ✓ Cardiomegaly ✓ Cardiac dysfunction Aging? Obesity? Hypertension? Immunometabolism dysfunction?	Mouse (58)
Dahl salt-sensitive and ZSF1 models	✓ Obesity ✓ Aging ✓ Hypertension ✓ Metabolism dysfunction ✓ Cardiac dysfunction Systemic inflammation? Renal dysfunction?	Rat (59)

*(**v**) indicates experimental animal model that recapitulate HFpEF aspects. (?) indicates that the experimental animal model has not been studied for the indicated aspect. FPR2KO, formyl peptide receptor 2 knock out; Ob/Ob, obese mouse; db/db, db gene knock out, responsible for leptin receptor; ZF/ZDF, Zucker fatty/Zucker diabetic fatty; SKO, seipin knock out; ZSF1, Zucker spontaneously fatty hypertensive heart failure F1 hybrid*.

### Sterile Inflammation Resolution in Cardiac Repair and Suboptimal Inflammation in Heart Failure With Preserved Ejection Fraction

**Figure 2:**
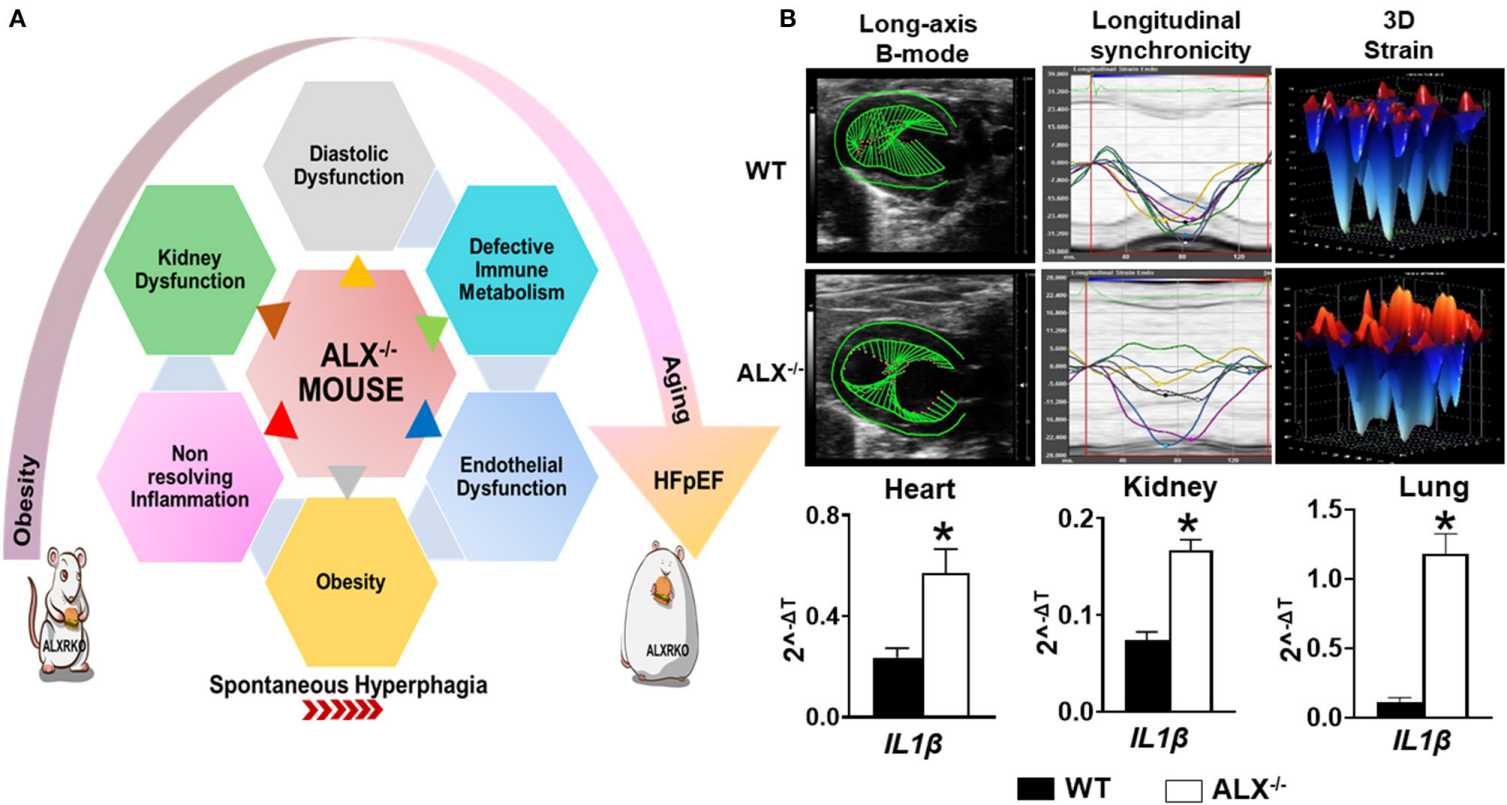
Resolvin D1 and lipoxin receptor-deficient mice (FPR2/ALXKO) complement the criteria of HFpEF with profound age-associated endothelial dysfunction and inflammation in the spleen, heart, and kidneys with signs of chronic inflammation. **(A)** Left panel indicates the heterogeneous, multidimensional, and multiorgan metabolic defects that contribute to HFpEF. **(B)** Right panel indicates the dysfunction of the resolution receptor FPR2/ALX in mice that develops spontaneous obesity with diastolic dysfunction along with limited changes in systolic function when obesity is superimposed on aging as signs of preserved ejection fraction. Suboptimal inflammation indicated with profound activation of IL1β gene expression in the heart, kidneys, and lungs in FPR2/ALXKO mice exemplifies the age-associated cardiac strain dysfunction, myocardium dissynchronicity, and amplified inflammation. *p < 0.05 compared to control WT group.

In response to an ischemic event, unresolved inflammation after cardiac injury is one of the common causal contributors in HFrEF (60). In the last two decades with high incidence of metabolic defects, it has been proven that inflammatory markers and array of cytokines may improve HF risk stratification and are considered predictors of HFpEF incident (61, 62). To date, different studies are ongoing to understand the cellular and molecular mechanisms by which the inflammatory response ends up in sustained inflammation that triggers unresolved inflammation leading to HFpEF. Recent preclinical studies in mice dissect the macrophage's role in HFpEF therapy and open up previously unexplored treatment options (63). Post-MI, macrophage temporal expression during the initiation of inflammation is a critical determinant of cardiac repair. However, we must note that progression and resolution of inflammation are overlapping and active processes aimed to control undesirable long-term inflammation. These overlapping processes are a hallmark of macrophage function and characterized by the production of the specialized proresolving mediator (SPM) biosynthesis. A recent report suggests that the deletion of resolution sensor ALX/FPR2 develop age-related obesity and diastolic dysfunction in mice with HFpEF (Figure 2) (52). After cardiac injury, ALX/FPR2 null mice induced macrophage dysfunction thereby lowering SPMs biosynthesis in the infarcted myocardium with defective activation of leukocytes in the spleen and heart (28, 52). Thus, macrophages are key in SPM biosynthesis from one side to resolve inflammation but also perform as master regulators during inflammation if they are pre- or overactivated before cardiac injury. Over the years, the dichotomy of M1/M2 macrophage spectrum is limited to describe macrophage function and amalgam of these key players required for myocardium homeostasis (64). During the resolution phase, reparative (M2) macrophages secrete high levels of TGFβ1, which drives the transcription of alpha smooth muscle actin (α-SMA) in the resident fibroblasts. Hence, it leads to the stiffness of the heart due to increased fibrosis, which reduces diastolic relaxation if it is over sustained (65). The explanation of macrophage dysfunction is described by many reports as pointing once inflammation is activated; a crosstalk is initiated between leukocytes, especially macrophage, and different types of cells such as endothelial and fibroblast cells, in a paracrine relationship. After cardiac injury, the crosstalk of activated macrophages and fibroblasts is necessary for ECM generation and reparative scar formation indicative of safe clearance of inflammation (66, 67). These findings emphasize the macrophage role in physiological or pathological inflammation in the development of HFpEF especially the source of a ripple or stream effect in the disease pathogenesis depending on the time, the location, and the milieu.

The other side of HFpEF is systemic inflammation and is an inherent phenotype in HFpEF patients. In clinical trials, in a group of patients with HF, it was confirmed that TNFR 2 plasma levels were significantly associated with the degree of diastolic dysfunction in patients with HFpEF but not HFrEF (68, 69). Besides myocardium inflammation, several cell types and tissues could contribute to systemic inflammation in patients with chronic HF as leukocytes and tissue macrophages and endothelial cells (70). The precise root cause of multiorgan inflammation is unclear, however, postulated from the intrinsic lifestyle factors such as imbalanced diet, sleep, and activity. Whether inflammation is a cause or consequence in the progression of the disease is still under investigation. Recent reports indicate that abdominal obesity is associated with an increased risk of all-cause mortality in patients with HFpEF (93). Aging, hypertension, diabetes, kidney, and endothelial dysfunction are a cluster of non-cardiac metabolic risk factors that are causal contributors to suboptimal inflammation with consequent HFpEF and further mortality in HFpEF patients (71, 72). Based on these facts, it is becoming clear that the presence of suboptimal or low-grade inflammation related to metabolic defects is behind the delay or worsening the start/progression of resolution of inflammation in HF. The sensor of resolution of inflammation as FPR2 receptors and SPMs were partially studied in experimental models of obesity superimposed on aging or “obesogenic aging.” However, Toll-like receptor (TLR) pathways have not been studied in animal models with multiple risk factors. TLRs are one of the main innate immune receptors that serve as mediators of sterile inflammatory conditions (73, 74). Activated immune cells release damage-associated molecular patterns (DAMPS) that activate cardiomyocytes that switch on to TLR4-mediated cardiac apoptosis (75).

HFrEF patients are prone to pathogen-directed systemic inflammation like sepsis (76). There are some contradicting clinical reports suggesting that obesity can prevent mortality rate in septic patients with chronic heart failure (CHF) (77) with limited supportive evidence and explanation. The paradigm is how obesity influences the immune response of septic patients, by increasing the whole proinflammatory response, but at the same time improving survival rate (78). One common explanation of this outcome is that lipid mediators/metabolites act as scavengers in circulatory blood and bind to endotoxins/leukocyte receptors and perform as antagonists (79). Future prospective studies should quantify adipose tissue at or before sepsis diagnosis to accurately identify true association between obesity and septic HF etiology (80). The presence of suboptimal inflammation plays a pivotal role in the initiation/progression of the disease. From this perspective, here, we emphasized the evolution of HFpEF from the concept of one-dimensional to multidimensional view where suboptimal inflammation serves as the hallmark in HFpEF progression due to the cluster of metabolic defects.

### Immune Metabolism and Macrophage Dysfunction

Based on the integrative function of leukocytes and metabolism, we discovered that splenic leukocytes dysregulation due to obesity or age-related unresolved inflammation is a primary mechanism of HFpEF in obesogenic aging. Immune cells, particularly leukocytes, operate for the resolution of inflammation and tissue repair. Cardiac injury site operates in the inflammation-resolution program with a supply of necessary energy nutrients and oxygen demand to accomplish processes of phagocytosis and microbial killing (81, 82). At the cellular level, if there is a metabolic defect, the phagocytosis mechanism would be certainly affected, which is common in obesity (83), in addition to the fact that changes in nutritional status impact immune cell metabolism and function due to Treg cells, which induces a significant shift in immune cell populations and cytokine production toward a proinflammatory state (84). Recent data showed that a resolution sensor FPR2/ALX plays a central role in metabolic homeostasis interaction with immune metabolism. Intriguingly, FPR2/ALX is essential for safe clearance of inflammation postcardiac injury (85, 86). In fact, FPR2/ALX receptor agonists lipoxins and resolvins, known as specialized proresolving mediators, decreased after ischemia in resolution receptor-deficient mice. In addition, the pharmacological inhibition of FPR2/ALX by WRW4 impaired leukocyte recruitment and elicited non-resolving inflammation in acute HF by limiting leukocyte mobilization to the injured site (87). Furthermore, in the absence of ischemic injury, young and aging mice show signs of metabolic defects after deletion of the FPR2/ALX receptor (Figure 2). Moreover, the immune response coordination of the leukocyte reservoir from the spleen to the heart (splenocardiac axis) was disrupted in these mice because of macrophage dysfunction (28, 52). Recent reports indicate that infiltrating macrophages undergo metabolic re-programming in cardiac repair process to increase oxidative phosphorylation post-MI (88–90). Increased oxidative phosphorylation in addition to fatty acid synthesis and oxidation is a signature of reparative (M2) phenotype (91). In the clinical setting, HF failure is marked with the presence of unresolved inflammation after MI with multiple risk factors. This notion regained interest in the last three decades after the discovery of the resolution of inflammation concept where elevated levels of tumor necrosis factor-α (TNF-α) were observed in HFpEF patients (92). As a therapeutic approach, blockade of the inflammatory cytokines and chemokines turned out as a negative outcome in the clinical settings. However, modulating the fate map of leukocytes, particularly macrophages, representing up to 50% of all cell types within the hypertrophic obese adipose tissue of mice and humans, appear to be potentially successful procedures (93–96). Over the years, there has been a growing recognition on how initiation, activation, and programming of the immune cell lead to the on/off of the inflammatory response in cardiac tissue repair. Since immune cell activation and its phenotypes are related to the metabolism, studies are focusing in defining the metabolic plasticity of immune cells after an injury. Research reflects the complexity of immune-metabolic signaling, networks, and the cellular and molecular events that can determine either the return to homeostasis or failure of the heart. The impact of metabolic disturbance, due to obesity with defective immune metabolism, misalign the activation of immune cells by enabling them to access the extracellular environment. From one side, in obese mice, the fat mass alters the integrity of the architecture of the immune tissue, thereby, impacting leukocyte roles postcardiac injury (97, 98). From another side, emerging evidence shows that activation of the immune response in the setting of obesity is activated through free fatty acid (FFA) metabolic signals by different molecular signaling pathways leading to stimulation of critical inflammatory signaling cascades (99). In fact, *in vivo* studies have shown that increasing plasma FFA levels activate NF-κB in human skeletal muscle, which leads to an increased expression of proinflammatory cytokines and elevated circulating levels of MCP-1 (100). Also, circulating FFAs impair insulin sensitivity through binding to the plasma membrane receptor Toll-like receptor 4 (TLR4) in tissues of obese animals, resulting in the activation of signaling proteins, such as inhibitor of nuclear factor-κB (IκB) kinase (IKK), c-Jun N-terminal kinase (JNK), and mitogen-activated protein kinase (MAPK), that negatively dysregulate the metabolic axis of macrophage polarization favoring chronic inflammation (101, 102). Overall, physiological studies strongly support a reciprocal relationship between the FFAs and respective receptors (FFARs) that helps in regulating the metabolic–inflammatory axis in HFpEF.

### Endothelial Dysfunction in Heart Failure With Preserved Ejection Fraction

Chronic inflammation is strongly linked to endothelial dysfunction (103, 104), since inflammatory signals activate the endothelial cells and fibroblasts resulting in subsequent concentric cardiac remodeling and dysfunction (105). Endothelial dysfunction is portrayed as one of the cellular mechanisms underlying HFpEF syndrome. In fact, the decline in endothelial NO bioavailability is observed in 90% of patients with HFpEF along with the decrease in intracellular cGMP and protein kinase G activity kinases that contribute to LV filling pressures and triggers the delay of myocardial relaxation (106, 107). Recent studies observed that the microvascular endothelial dysfunction is primed essentially by activated macrophages due to suboptimal inflammation (52, 108, 109). In obese animal models, targeting CCR2 helps to avoid accumulation of macrophages in the vascular wall thereby reducing endothelial dysfunction and oxidative stress (109, 110). Moreover, in experimental model of aging, it has been reported that endothelial senescence and inflammation are associated through the senescence-associated secretory phenotype. Age-related inflammation contribute to the development of HFpEF early stage marked by hemodynamic and structural changes evolving to a typical HFpEF phenotype (111). Besides aging and inflammation inducing endothelial dysfunction, the deficiency of protease-activated receptor 2 (PAR2) is associated with extracellular matrix remodeling, which might be an alternative contributor to HFpEF pathophysiology (112). In addition, emerging explanation for endothelial dysfunction in HFpEF is the endothelial metabolic reprogramming. Recently, it has been shown that the impairment of silent mating type information regulation 2 homolog (SIRT3)-mediated endothelial cell metabolism may lead to a disruption of communications between myocyte adjacent cells and coronary microvascular rarefaction (113). In this context, a model of cardiac metabolic disorder due to the dysfunction of ALX receptor, essential for the resolution of inflammation in cardiac repair (114), shows a pronounced cardiorenal endothelial dysfunction in the mice along with diastolic dysfunction and preserved EF (52) (Figure 2). In an age-related study, ALX/FPR2KO mice decreased protein and gene expressions of eNOS and CD31 in the heart and kidneys with lower distribution of both endothelial markers in tissues compared with age-matched controls (28, 52). In addition, recent findings highlighted an improved endothelial function in Zucker spontaneously fatty hypertensive heart failure F1 hybrid (ZSF1) obese rats, a recent model qualifying for HFpEF studies including hypertension and diabetes. In this study, supplementation of the NAD^+^ precursor nicotinamide (NAM) elicited an antihypertensive effect correlated with improved endothelial function, as indicated by an enhanced vasodilatory response to acetylcholine in isolated aortic rings (59) (Table 1).

## Prevention and Treatment Perspective

**Figure 3:**
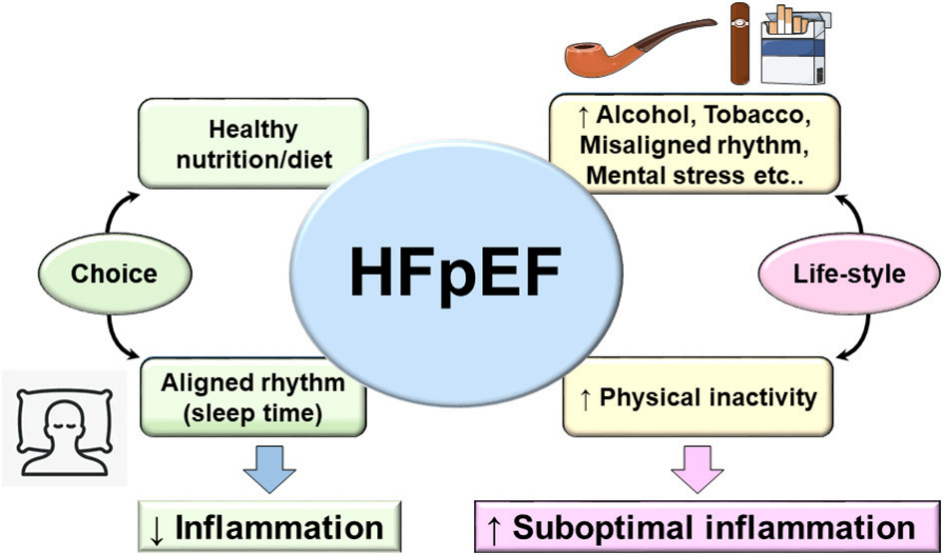
Fundamental and lifestyle-associated prime factors that control inflammation (left arm of figure) in contrast to risk factors that drive suboptimal inflammation in HFpEF and integration with lifestyle-associated molecular patterns (LAMPs). The main four aspects of daily life to consider as prime strategy for prevention of HFpEF (diet/nutrient, sleep/wake up rhythm, exercise, and choice of active life and limited sedentary lifestyle). Adoption of optimal age-related lifestyle would be helpful to balance the genesis of LAMPs in cardiovascular disease that will help to limit the progression to heart failure.

The recent PARAGON clinical trial resulted in FDA approval to the Entresto drug, a sacubitril/valsartan combination, as the first treatment for HFpEF patients. However, due to the diversity and complexity of HFpEF patient phenotype, a large proportion of HFpEF patients will remain without meaningful treatment options considering the exclusion criteria in the actual treatment (115). This brings insights into the fundamental foundation for HFpEF treatment that remains supported by primary preventive treatment that focuses on lifestyle. As of today, no treatment is patient specific that target multiorgan and system-based approach due to heterogeneous pathology. Based on sedentary lifestyle, new terminology emerged termed as lifestyle-associated inflammatory diseases and their corresponding immune-stimulatory lifestyle-associated molecular patterns (LAMPs) (116). LAMPs are third generation after classical pathogen-associated molecular patterns (PAMPs) as lipopolysaccharide (LPS) or infection and damage-associated molecular patterns (DAMPs) as serum amyloid A or TLRs, known to induce inflammation-related disease. However, LAMPs impair the sterile inflammation due to our lifestyle-associated risk factors. As a self-choice avoiding known risk factors like sleep cycle regulation (117) (respecting body clock), diet/nutrient [healthy food with eicosapentaenoic acid (EPA) and docosahexaenoic acid (DHA) food intake], and exercise (118) (maintaining active lifestyle) might entail a balanced metabolism and help in delaying the inevitable decline in various body systems and physiological processes of aging. For total health, it is obvious to avoid or limit smoking (both passive and active smoke), and control psychological stress and alcohol intake (Figure 3). Thus, third-generation novel LAMPs are manageable and should be considered strongly to reduce the burden of prevalence of HFpEF in obesogenic aging (119, 120). Molecular and cellular mechanism of LAMPs is warranted after extensive research on PAMPs and DAMPs.

## Conclusion

Like every area of life sciences, change is not permanent, and likewise, the HF pathophysiology evolved from one-dimensional homogenous pathology to heterogeneous multiorgan dysfunction particularly in obesity/metabolic syndrome superimposed on aging. From a prevention perspective, primary focus on diet/nutrition intake (quality/quantity), sleep–wake up cycle (circadian rhythm), and physical activity (exercise/sedentary lifestyle) are key determinants of total health and specifically for cardiovascular health (Figure 3). Recent ongoing clinical trials for HFpEF are targeting metabolic diseases like type 2 diabetes/insulin resistance/hyperglycemia with improvement in cardiovascular outcome that underline the strength of metabolic disorder and HF-related suboptimal inflammation. However, the major missing point in HFpEF is the multitude of phenotypes in HF syndrome of which half of the patients are either obese or aging or both, which make the clinical cases deeply complicated; thus, innovative animal model and treatment strategy are extensively warranted to study lifestyle-associated molecular patterns (LAMPs).

## Author Contributions

GH conceptualized the outline, edited the multiple drafts, prepared/edited the figures, and approved for submission. BT prepared the first draft and re-edited after the input from GH. All authors contributed to the article and approved the submitted version.

## Conflict of Interest

The authors declare that the research was conducted in the absence of any commercial or financial relationships that could be construed as a potential conflict of interest.

## Publisher's Note

All claims expressed in this article are solely those of the authors and do not necessarily represent those of their affiliated organizations, or those of the publisher, the editors and the reviewers. Any product that may be evaluated in this article, or claim that may be made by its manufacturer, is not guaranteed or endorsed by the publisher.
